# Death of Doctor W. W. Dawson, M. D.

**Published:** 1893-05

**Authors:** 


### American Medical Association.

### NECROLOGY.

### W. W. Dawson, M. D.

It is a sad duty to record the death in Cincinnati, February 16th, of Dr. Wm. W. Dawson, M.D. Dr. Dawson for about a third of a century was one of the leading physicians in this city—Cincinnati. He was one of the prominent men of the medical profession in this country. From 1860 to the time of his death he was a medical teacher, having a connection as one of the professors in the Ohio Medical College, and during a large share of this time he was a member of the surgical staff in Cincinnati and other hospitals. He has held almost every prominent position within the gift of his medical brethren. He has been President of the American Medical Association, and was one of its trustees at the time of his death. He was at one time President of the Cincinnati Academy of Medicine, and subsequently President of the Ohio State Medical Society. He was throughout his professional life a successful and popular physician and surgeon. His diagnostic ability was of a very high order, and his skill as a surgical operator had no superiors and very few equals. His bearing to his professional brethren was of the highest type, always courteous and the very soul of honor. His personal friends numbered thousand. He was one of nature's noblemen. Who shall come to take his place?

